# HNF1A gene mutations and premature ovarian failure (POF): evidence from a clinical paradigm combining MODY 3 and POF

**DOI:** 10.1007/s42000-024-00529-y

**Published:** 2024-02-05

**Authors:** P. Xekouki, A. Konstantinidou, C. Tatsi, A. Sertedaki, N. Settas, D. Loutradis, G. P. Chrousos, C. Kanaka-Gantenbein, C. Dacou-Voutetakis, A. Voutetakis

**Affiliations:** 1 Endocrine and Diabetes Clinic, University General Hospital of Heraklion, Medical School, University of Crete, 71500 Heraklion, Crete, Greece; 2 1st Department of Pathology, Unit of Perinatal Pathology, School of Medicine, National Kapodistrian University of Athens, Athens, Greece; 3 Division of Endocrinology, Diabetes and Metabolism, “Aghia Sophia” Children’s Hospital ENDO-ERN Center for Rare Paediatric Endocrine Diseases, First Department of Pediatrics, Medical School, National and Kapodistrian University of Athens, Athens, Greece; 4 1st Department of Obstetrics and Gynecology, Alexandra Hospital, Athens University Medical School, Lourou 4-2, 115 28, Athens, Greece; 5 Department of Pediatrics, University General Hospital of Alexandroupolis, Democritus University of Thrace, Alexandroupolis, Greece

**Keywords:** HNF1A mutations, Premature ovarian failure, MODY diabetes, Ovary, Premature menopause

## Abstract

Premature ovarian failure (POF) defines the occurrence of ovarian failure prior to the age of 40. It occurs in one out of 100 women but is very rare before age 20 (1:10,000). Maturity-onset diabetes of the young (MODY), caused by mutations in the *HNF1A* gene, is also a rare disorder; all types of MODY account for 1–2% of adult diabetic cases. These two rare nosologic entities coexisted in an adolescent girl evaluated for delayed puberty. Although this combination could represent a chance association, an interrelation might exist. We examined HNF1A expression in human fetal and adult ovaries by immunohistochemistry using a polyclonal HNF1A antibody. HNF1A protein was expressed in both the fetal and adult human ovaries. Based on these findings, we hypothesize that HNF1A participates in ovarian organogenesis and/or function and that mutations in the *HNF1A* gene might represent another molecular defect causing POF, possibly in combination with other genetic factors. The study underlines the importance of rare clinical paradigms in leading the way to elucidation of the pathogenetic mechanisms of rare diseases.

## Introduction

Premature ovarian failure (POF) or insufficiency (POI) is defined as the development of hypergonadotropic ovarian failure prior to age 40. It occurs in 1:100 of all women, 1:1000 of women prior to age 30, and 1:10,000 of females aged less than 20 years [1, 2]. POF might be manifested as primary or secondary amenorrhea, irregular menses, delayed puberty, infertility, and/or premature menopause. POF could be the result of either fewer follicles, formed during ovarian ontogenesis, or an increased rate of follicle loss. Alternatively, while the follicles are present, they are unresponsive to gonadotropins [3].

POF is a heterogeneous disorder caused by genetic (~ 50%), autoimmune (~ 10%), or iatrogenic factors, and it may be encountered either as an isolated pathologic entity or as part of a syndrome [1, 2]. In the absence of oophorectomy, chemotherapy, irradiation, or chromosomal aberration, the pathogenesis of POF remains unknown in approximately 90% of cases. About 30% of POF cases are familial, this pointing to a genetic predisposition in a significant number of affected individuals [1, 4, 5]. Although several candidate genes have been implicated in POF (*BMP15*, *FOXL2 NOBOX*, and others), the underlying molecular defect is yet to be determined in the majority of cases [5–8].

MODY 3 is caused by mutations in the hepatocyte nuclear factor 1A (HNF1A) and constitutes a rare type of monogenic diabetes, all forms of MODY accounting for 1–2% of diabetes cases in adulthood [9, 10].

The combined nosology of POI and MODY 3 diabetes was reported by our group in 2011 at the Annual Μeeting of the European Society of Pediatric Endocrinology (ESPE), the question being raised as to whether or not these two entities represent a coincidental event or are interrelated [11]. Recently, Alvarez et al. reported a case in which the same combination of nosologies was presented, raising the same question. In their case, whole exome sequencing (WES) disclosed a mutation in the HNF1α gene, but no variants in genes related to POI were detected [12]. The latter report prompted a re-evaluation of our initial case, and the results are herein presented.

## Case description

Our patient, a young female, was initially examined at the age of 14^2^/_12_ years because of lack of pubertal development and slightly elevated fasting blood glucose. Physical examination showed a girl of tall stature, with normal body mass index (BMI), without morphologic stigmata, and of normal intelligence.

She was born after an uneventful pregnancy and had a birth weight of 4350 g. Family history revealed that the mother, aged 38 years, had regular menses and no hyperglycemia. The father, aged 44, had recently been found to have a fasting hyperglycemia of 130 mg/dL. The maternal grandmother has had elevated blood glucose (130–150 mg/dL) since the age of 57.

With estrogen and progesterone replacement therapy, pubertal development progressed normally and menses occurred regularly in our patient. Her final height was 173 cm with a BMI of 20.6 kg/m^2^. Her hyperglycemia was adequately controlled by means of an appropriate diet combined with an oral hypoglycemic agent.

Gonadotropins, prolactin, adrenal steroids, insulin, thyroid hormones, and routine biochemistry were determined using the appropriate methodology. Blood glucose and insulin values were determined via an oral glucose tolerance test (75 g) performed after an overnight fast.

DNA was extracted from peripheral blood leukocytes using the QIAamp DNA Blood Mini Kit (QIAGEN, Germany). The ten exons and their flanking intronic sequences of the *HNF1A* gene were PCR amplified. Sequencing was carried out bidirectionally on a genetic analyzer (ABI 3100, Applied Biosystems, USA).

In an attempt to interpret the coexistence of POF and HNF1A mutation, immunohistochemical studies in human ovarian tissue were conducted. Expression of the HNF1A protein was examined by immunohistochemistry using a rabbit polyclonal antibody that binds to human HNF1A (HNF1 NBP1-33,596 Novus Biologicals, diluted 1:200, pretreated with citrate buffer pH = 6, prior to primary antibody addition). The examined samples consisted of human fetal ovarian tissue obtained at autopsy from fetuses of 23, 32, and 40 weeks of gestation, as well as ovarian tissue from surgical specimens of adult premenopausal women, aged 44–48 years, who underwent surgery for uterine fibromyomas or ovarian cysts. Liver tissue was used as a control sample.

Written informed consent was obtained from the patient for publication of this case report and any accompanying images.

## Results

The basal values of gonadotropins in our patient were high on several occasions (at age 14: FSH 121 mIU/mL and LH 24 mIU/mL), confirming hypergonadotropic hypogonadism. The levels of prolactin, thyroid hormones, lipids, liver enzymes, and adrenal androgens as well as routine biochemistry were normal (Table 1).

**Table 1:** Results of pertinent initial studies. Reference values in parenthesis

FSH	121 mIU/mL (2.5–10.2)		
LH	24.3 mIU/mL (1.9–12.5)		
TSH	2.2 µU/mL (0.5–4.7)		
T4	113 nmol/L (80–140)		
Prl	22 ng/mL (28–29)		
Fasting blood glucose	90–140 mg/dL (80–110 mg/dL)		
Oral glucose tolerance test (1.75 g/kg body weight), amount given: 75 g		Glucose (mg/dL)	Insulin (µU/mL)
	0′	90	4
	30′	191	11
	60′	234	16
	120′	187	6
Islet cell antibodies	Negative		
Anti-thyroid antibodies	Negative		
Karyotype	46,XX		
Liver enzymes	SGOT, 21 IU/L (11–38); SGPT, 28 IU/L (11–43); γGT, 10 IU/L (8–15)		
Blood urea nitrogen	36 mg/dL (10–50)		
Creatinine	0.7 mg/dL (0.6–1.4)		
Total cholesterol	149 mg/dL (130–200)		

An oral glucose tolerance test showed hyperglycemia and insulinopenia (Table 1). The ICA, anti-Tg, and anti-TPO antibodies were negative, and the karyotype was 46,XX. Fasting blood glucose values ranged from 90 to 140 mg/dL.

Ultrasonography of the ovaries showed very small ovaries on different occasions.

DNA analysis revealed *HNF1α* gene mutation in heterozygosity (c.481G > C, p. A161P). Sequencing a number of genes related to POI (*GDF9*, *BMP15*, *FOXL2*, *FOXE1*, *and NOBOX*) revealed the following: a heterozygous missense variant p.F517L (rs2699503) in the *NOBOX* gene, a homozygous variant in the promoter region of the *BMP15* gene c.-9C > G (rs3810682), and a homozygous synonymous variant p.T149T (rs254286) in the *GDF9* gene. Fonseca et al. [13], who studied the *BMP15* gene c.-9C > G (rs3810682), found that the c.-9G allele was related to increased *BMP15* gene transcription. The latter is a factor contributing to the development of POI.

**Fig. 1:**
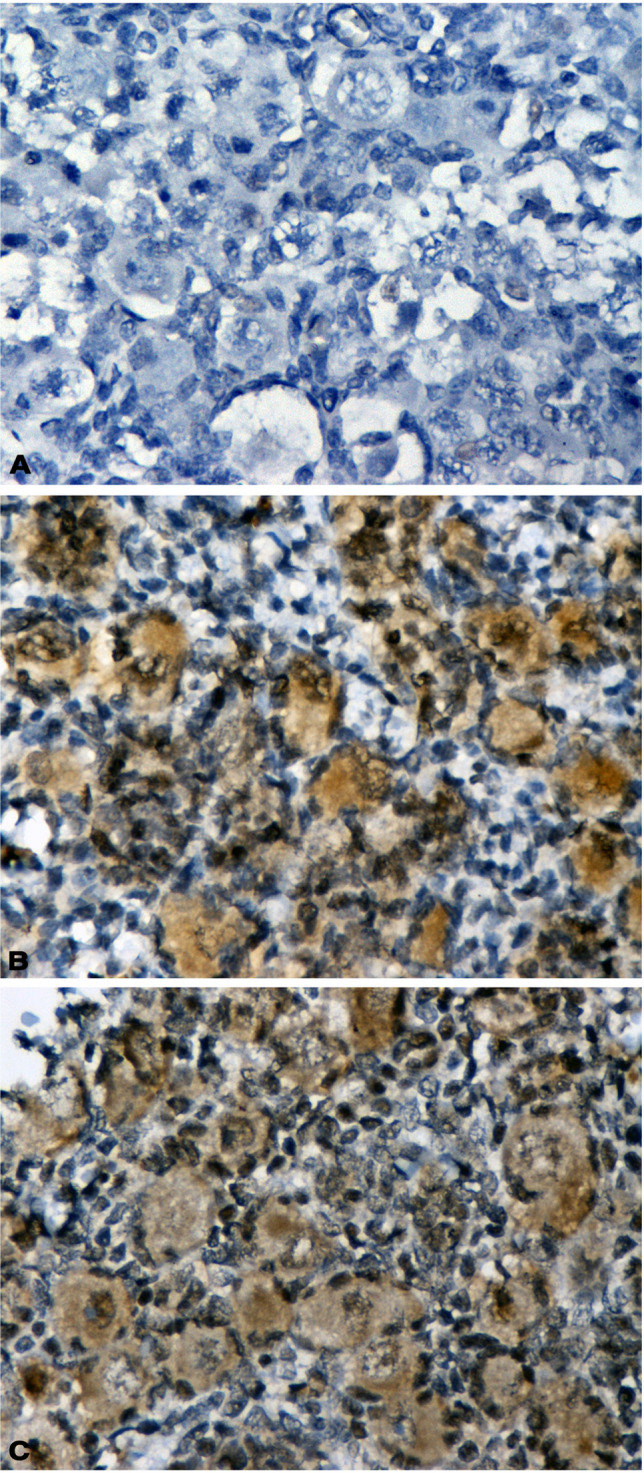
Immunohistochemical staining indicating HNF1A expression in fetal ovaries (HNF1A X 400). **A** No expression at 23 weeks of gestation. **B**, **C** Positive expression in the primordial follicles at 32 and 40 weeks of gestation

**Fig. 2:**
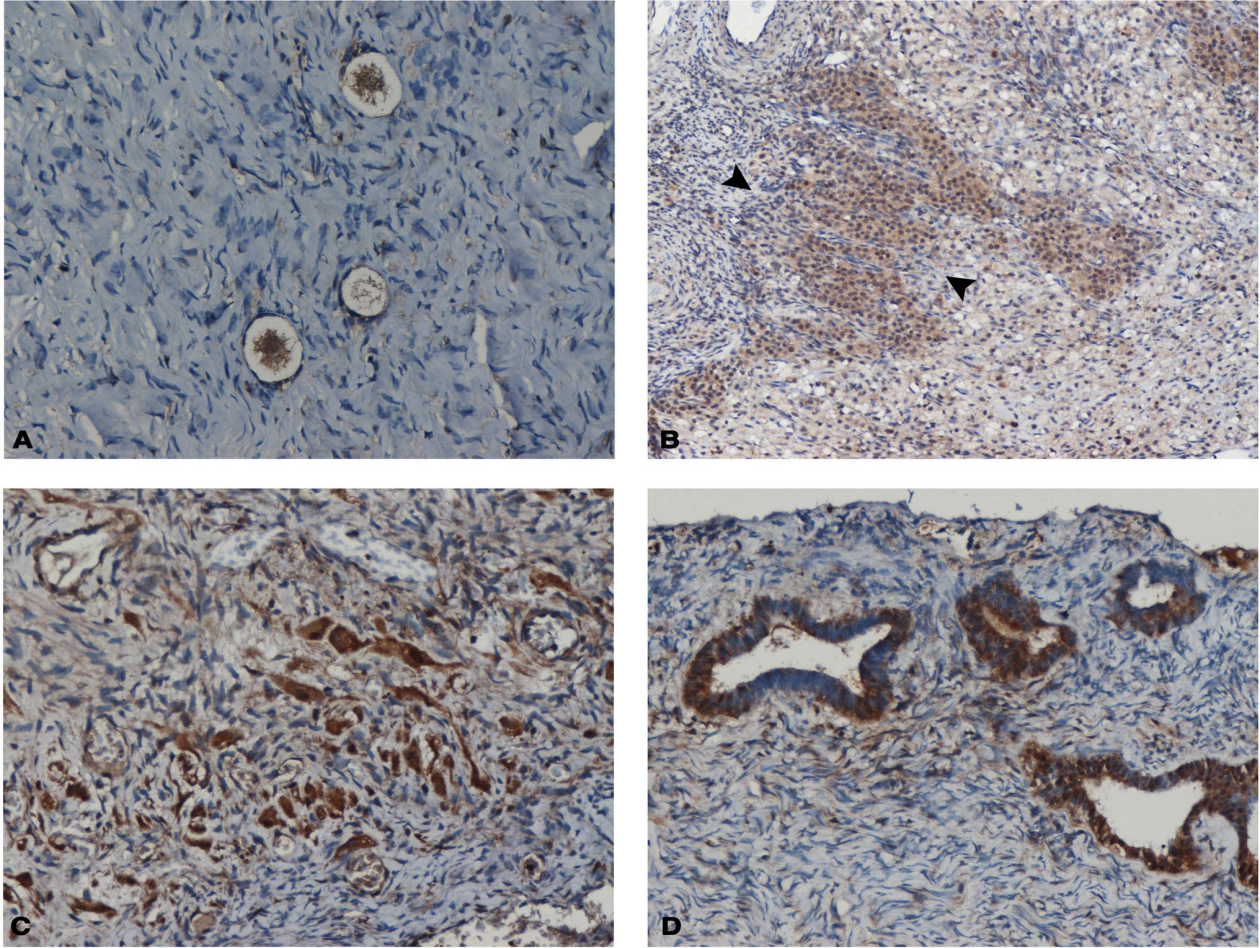
Immunohistochemical staining indicating HNF1A protein expression in the ovaries of adult premenopausal women. **A** Primary ovarian follicles (× 200). **B** Corpus luteum of menstruation: faint HNF1A expression in the thick inner layer of large granulosa-lutein cells and more intense expression in the outer thinner layer of smaller theca-lutein cells (× 100)—between arrow heads. **C** Luteinized stromal cells (× 200). **D** Epithelial inclusion glands of the ovarian cortex (× 200)

Immunohistochemistry showed that the HNF1A protein is expressed in human fetal ovaries from late gestation and onwards, being localized in the cytoplasm and nucleus of primordial follicles (Fig. 1A–C). Expression of the protein was maintained in adult premenopausal women at various sites of the ovaries (Fig. 2A–D), including primordial, primary, and maturing follicles, theca-lutein and granulosa-lutein cells of the corpus luteum, luteinized and fibroblastic stromal cells, and various epithelial parts (surface epithelium, epithelial inclusion glands, and *rete ovarii*, as well as the epithelium lining the fallopian tube).

## Discussion

In the present case report, we describe a case of POI coexisting with MODY 3, caused by *HNF1α* gene mutation.

Ovarian dysfunction could theoretically be the result of diabetes [7, 14]; however, this is not likely in our case since POI started quite early (prior to the age of 13 years), after which the patient developed asymptomatic hyperglycemia. Thus, POI should not be considered a *phenomenon* secondary to diabetes. The general condition of our patient was good, and somatic growth and mental development were normal.

As mentioned previously, POI can manifest as an isolated nosological entity. It can, on the other hand, also be encountered in association with diverse types of disorders (e.g., hearing loss) as well as Perrault syndrome, pituitary hypoplasia, and peripheral neuropathy, some of which have been attributed to molecular defects in various genes [4, 15–18].

We screened the literature for *HNF1A* gene expression in the ovaries, which, if present, could offer a hint with regard to associated nosologies. Pertinent information is limited and, at present, restricted to animals. As shown by Lee et al., *hnf1a* null female mice present an infantile uterus with intense hypoplasia, especially of the myometrium. Histology, however, revealed that folliculogenesis was ongoing in the ovaries of the *HNF1A*^(−)/(−)^ animal and was indistinguishable from that of controls [14].

Additionally, Huang et al., using immunoprecipitation and western blot, reported that HNF1A, 1B, and 3Β proteins were detected in both the liver and gonads in tilapia [23]. Huang and Weng, in a review article, noted that several members of the HNF family have been detected in teleost gonads [19]. Thus, expression of HNFs in the gonads of tilapia suggests that multi-HNFs may create a cascade that regulates gonadal physiology in bony fish.

Hypothesizing that the HNF1A protein might be expressed in the human ovary at specific developmental stages and, when mutated, could lead to ovarian dysfunction, we performed immunohistochemical staining on ovarian tissue from human fetuses and adult women. Ovarian tissue of pubertal age was not available. HNF1A protein was detected in the primordial follicles of mature fetuses at a late gestational age and in a wide range of histologic structures in the ovaries and fallopian tubes of adult premenopausal women, being particularly frequently expressed in cells with steroidogenic capacity and responsiveness to gonadotropins.

We therefore assumed that in our patient, the ovarian dysfunction might be related to altered *HNF1A* gene expression. The patient’s mother, although a carrier of the same molecular defect in the *HNF1A* gene, had neither diabetes nor POF. This is not necessarily contradictory since asymptomatic carriers of molecular defects have been reported in other genetic disorders, including MODY 3 [9, 15, 20, 21].

An alternative explanation for the combined nosology in our case could be the potential coexistence of variants in POI-related genes (*NOBOX* and *BMP15*).

In the case of Alvarez et al., the ovaries had a normal sonographic appearance, somewhat resembling that of an *hnf* null mouse [12], whereas in our patient, the ovaries were hypoplastic, possibly as a result of variants in the POI-related genes (*BMP15*–*9* G/G: in our cohort, 15% in POI patients, 0% in our controls, and 5% in 1000GP (p 0.009)).

Further studies are required to prove or disprove this hypothesis. Nevertheless, the data herein provide evidence that *HNF1A* is expressed in the human ovary as from the fetal stage, in certain cases, deranged expression, either independently or combined with other molecular derangements or adverse environmental factors, might lead to premature ovarian failure [22].

The current paper emphasizes the importance of extremely rare clinical paradigms in leading the way to clarification of the pathogenetic mechanisms of rare disorders.

## Conclusion

Most likely, these two nosologic entities are genetically interrelated. The knowledge that *HNF1A* is expressed in the ovaries might constitute an explanation for the combination. Furthermore, these findings should alert physicians managing patients with MODY 3 to screen for hypogonadism early in the course of the disease.
